# Addressing NCDs: Penetration of the Producers of Hazardous Products into Global Health Environment Requires a Strong Response: Comment on "Addressing NCDs: Challenges From Industry Market Promotion and Interferences"

**DOI:** 10.15171/ijhpm.2019.52

**Published:** 2019-07-03

**Authors:** Sally Casswell

**Affiliations:** SHORE & Whariki Research Centre, College of Health, Massey University, Auckland, New Zealand.

**Keywords:** Non-communicable Diseases, Private Public Partnerships, Conflict of Interest, Health Treaties, Industry Interference

## Abstract

Timely warnings and examples of industry interference in relation to tobacco, alcohol, food and breast milk substitutes are given in the editorial by Tangcharoensathien et al. Such interference is rife at national levels and also at the global level. In an era of ‘private public partnerships’ the alcohol and food industries have succeeded in insinuating themselves into the global health environment and their influence is seen in key recommendations regarding non-communicable disease (NCD) risk factors in United Nations (UN) reports. The absence of legally binding health treaties in these areas facilitates this industry engagement and the Framework Convention on Tobacco Control provides a valuable model to apply to control of other hazardous products.

The editorial by Tangcharoensathien et al^1^ warns about the challenges to the prevention of non-communicable disease (NCD) from four industries: tobacco, alcohol, food and breast milk substitute. It is only relatively recently that attention has been paid to the role of the industries, beyond that of tobacco, in simultaneously promoting unhealthy products and interfering in the uptake of effective policy. There is now a burgeoning research literature in this area and in 2018 the report from the United Nations (UN) Interagency Task Force described the interference by industry in their efforts to assist governments to put in place effective policy; the report stated that, while such interference from the tobacco industry had been long seen, country missions now see the equivalent from alcohol and food industry actors.^2^

The editorial provides examples of industry tactics from different countries concluding there are four main types: interfering with legislative process; using front groups to act on their behalf; questioning the evidence of harm and effectiveness of harm reduction interventions; and appearing responsible in the eyes of public, journalists and policy-makers. While providing valuable insights into what needs to happen at the national level to combat these tactics the editorial also refers to the need for action at the global level.

It should not be a surprise to any of us that these global industries are operating in this way. Transnational corporations share the same imperative - to maximise profits from sales of their products. This is the root cause of the conflicts of interest which exists across these four industries. In order to maximise profits, especially by expansion into middle-income countries with growing economies, the industries must avoid the implementation of effective policies, those which limit the over-supply, marketing and affordability of products, the UN system’s ‘Best Buys’ or most cost effective policies.

The authors perceive the tobacco industry as the most aggressive in terms of their interference in policy. However, the tobacco industry’s relative aggression may be a reflection of the greater constraints on their activity. This is a context in which governments and civil society have understood conflict of interest clearly and responded to the global threat to health by putting in place a legally binding treaty: the Framework Convention on Tobacco Control. This convention includes the very important clause (5.3) which specifically requires governments to avoid industry interference while developing and implementing their policies.

In comparison with tobacco the conflicts of interest which underpin the actions of the other industries have been less clearly understood and this has led to a very different positioning within the political environment both at national and global level. For example, the editorial refers to the alcohol industry as highly effective and well-organised in gaining access to the policy-making process, including by their close relationships with policy actors. Diageo, a major transnational alcohol corporation has reported to its shareholders the success of these measures saying they have achieved ‘more balanced regulatory outcomes’ as a result of their efforts.^3^

These industries frame themselves in similar ways and have succeeded in influencing policy by presenting themselves as part of the solution supported by their Corporate Social Responsibility activities. The solution is framed as one of individual responsibility drawing attention away from the hazardous properties inherent in the products and the central importance of promotion, supply and affordability to their spread and harmful use.^4^

The transnational alcohol corporations ubiquitous promotion of responsible drinking campaigns and their frame of the need to protect moderate drinkers from alcohol policy is a smokescreen which draws attention away from their reliance on the harmful use of alcohol which contributes a significant proportion of their sales and profits^5^ and underpins their conflict of interest.

At the global level the extent to which the food and sugar beverages and the alcohol industry have permeated the governance arena is astonishing. And while there is a growing literature describing the influence of the industries at national level^6-11^ there is less documentation of their impacts in the global health environment.

The encouragement of private public partnerships has provided a welcoming environment for self-promotion by these industries as being part of the solution and they have responded by strong engagement since NCDs emerged on the UN agenda. Examples include participation in the Global Health Council’s NCD Roundtable; PepsiCo and Coca-Cola contributed to policy recommendations to the 2011 UN High-Level Meeting (HLM) on NCDs and sponsored side-events at the UN.^12^ At the UN civil society hearings prior to the 2011 HLM^13^—the main opportunity for advocates to shape the final UN political declaration—representatives from food (including the International Food and Beverage Alliance) and alcohol industries (including Anheuser-Busch, SABMiller, and the Global Alcohol Producers group) were among the main representatives of ‘civil society.’^14^

In October 2017, the World Health Organization (WHO), along with the government of Uruguay, organised a global conference on NCDs in Montevideo, where governments endorsed the “Montevideo roadmap 2018-30” on NCDs. Examination of the early draft, written comments made during the consultation period, and the final road map show important changes to the document during the process and help identify key influencers and their effects. For example, taxation of sugar sweetened beverages and alcohol were included as possible options in the draft version but dropped from the final version (only tobacco taxation remained).^15^

In 2018 the recommendations from the UN HLM were again judged as having been very much compromised by input from countries representing the interests of the commercial determinants. A robust critique from global civil society pointed to the absence of strong language on implementing the Best Buys, which focus on taxation, regulation and legislation. This was described as a ‘glaring omission’ and was seen as reflecting ‘the interference and undue influence of health harming industries over a few countries who were prepared to shamelessly block progress for all.’^16^

The WHO Framework for Engagement with Non State Actors (FENSA) was endorsed by member states in 2016^17^ and addressed conflicts of interest in an era of increasing links between private enterprise and health; FENSA specifically excluded relationships with the tobacco industry but did not do so regarding the industries promoting alcohol, food and breast milk substitutes. The UN HLM 2018 report went further by recommending more frequent consultation by WHO with the alcohol industry and increased engagement was included in the WHO Programme of Work presented to the Executive Board in 2019. However, several countries questioned the proposed expansion of contact with the alcohol industry and following this, a few weeks later, for the first time, the WHO issued an Information Note to its staff providing clear guidelines limiting contact with the alcohol industry.^18^ This may mark a turning point in the global governance arena in relation to alcohol but the extent of any change remains to be seen.

In the meantime the warnings given by Tangcharoensathien et al^1^ of industry interference and the range of methods used to promote their interests remains a clarion call to those engaged at the national, regional and global levels in the struggle to improve the health and well-being of populations. There is no room for complacency until much greater awareness of the conflict of interest and regulation of industry practices through legally binding regulation is in place.

## Ethical issues

Not applicable.

## Competing interests

Author declares that she has no competing interests.

## Author’s contribution

SC is the single author of the paper.

## References

[1] Tangcharoensathien V, Chandrasiri O, Kunpeuk W, Markchang K, Pangkariya N (2019). Addressing NCDs: challenges from industry market promotion and interferences. Int J Health Policy Manag.

[2] World Health Organization. Preparation for the third High-level Meeting of the General Assembly on the Prevention and Control of Non-communicable Diseases, to be held in 2018 (Report by the Director-General). http://apps.who.int/gb/ebwha/pdf_files/EB142/B142_15-en.pdf. Accessed March 12, 2019. Published 2017.

[3] Diageo. Diageo Annual Report 2017. https://solutions.vwdservices.com/products/documents/ed10423a-de9a-4659-accc-9929cb728e0a/?c=gaGYNdzTkhDUVseiV4HYG24l3XwN2zxAPgv6N5ZGCVTYV1kjr4DaTm0S3YFbnKnd. Accessed May 30, 2018. Published 2017.

[4] Weishaar H, Dorfman L, Freudenberg N (2016). Why media representations of corporations matter for public health policy: a scoping review. BMC Public Health.

[5] Cuong PV, Casswell S, Parker K (2018). Cross-country comparison of proportion of alcohol consumed in harmful drinking occasions using the International Alcohol Control (IAC) study. Drug Alcohol Rev.

[6] Roache SA, Gostin LO (2017). The untapped power of soda taxes: incentivizing consumers, generating revenue, and altering corporate behavior. Int J Health Policy Manag.

[7] O’Kelly PM, Davies A, Greig AJ, Lee KK (2016). Obesity prevention in a city state: lessons from New York City during the Bloomberg Administration. Front Public Health.

[8] Babor T, Robaina K (2013). Public health, academic medicine, and the alcohol industry’s corporate social responsibility activities. Am J Public Health.

[9] Ferreira-Borges C, Endal D, Babor T, Dias S, Kachiwiya M, Zakeyu N (2014). Alcohol policy process in Malawi: Making it happen. Int J Alcohol Drug Res.

[10] McCambridge J, Hawkins B, Holden C (2014). Vested interests in addiction research and policy The challenge corporate lobbying poses to reducing society’s alcohol problems: insights from UK evidence on minimum unit pricing. Addiction.

[11] Hawkins B, Holden C, Eckhardt J, Lee K (2018). Reassessing policy paradigms: a comparison of the global tobacco and alcohol industries. Glob Public Health.

[12] Moscetti C, Taylor A (2015). Take me to your liter: politics, power and public-private partnerships with the sugar-sweetened beverage industry in the post-2015 development agenda. Wash Int Law J.

[13] World Health Organization. Civil society interactive hearing on noncommunicable diseases on 16 June 2011. https://www.who.int/nmh/events/2011/informal_hearing/en/index1.html. Accessed May 3, 2019. Published 2011.

[14] Stuckler D, Basu S, McKee M (2011). Commentary: UN high level meeting on non-communicable diseases: an opportunity for whom?. BMJ.

[15] Whitaker K, Webb D, Linou N (2018). Commercial influence in control of non-communicable diseases. BMJ.

[16] Lieberman A. UN meeting on NCDs falls short on hard commitments, civil society say. https://www.devex.com/news/un-meeting-on-ncds-falls-short-on-hard-commitments-civil-society-say-93547. Accessed May 2, 2019. Published 2018.

[17] World Health Organization (WHO). Framework of engagement with non-State actors. WHO website. http://www.who.int/about/collaborations/non-state-actors/A69_R10-FENSA-en.pdf. Accessed April 6, 2018. Published 2016.

[18] Torjesen I (2019). Exclusive: Partnering with alcohol industry on public health is not okay, WHO says. BMJ.

